# The use of individualised, media‐based sleep hygiene education for professional female footballers

**DOI:** 10.1002/ejsc.12247

**Published:** 2025-01-07

**Authors:** Julie Gooderick, Russ Clash, Harry Fisher, Neil Maxwell, Mark Hayes

**Affiliations:** ^1^ School of Sport University of Brighton Brighton UK; ^2^ School of Natural Sciences University of Kent Canterbury UK; ^3^ School of Allied Health University of Chichester Chichester UK

**Keywords:** health, recovery, relaxation, team sport

## Abstract

Sleep hygiene can be defined as practicing habits that facilitate sleep; poor sleep hygiene is common among elite athletes, and improving this can be one way to enhance sleep indices. Given the large inter‐individual variability of sleep, there is a need for further investigation into individualised sleep hygiene for elite female athletes, with consideration for the practical application of the method. Using a self‐controlled time series design with repeated measures, *n* = 16 professional female footballers completed a 9‐week study during mid‐season. Monitoring of sleep (actigraphy, self‐report) occurred at week 1, 4, 7 and 9—a control period occurred at week 2 and 3, and a subsequent intervention period occurred at weeks 5 and 6. Based on baseline sleep monitoring, media‐based messages were designed with the purpose of giving a singular sleep hygiene message; all participants received these individualised messages daily across the 2‐week intervention period at a standardised time of 8.00 p.m., with the intention of them actioning the sleep hygiene point. One‐way analysis of variance with repeated measures was conducted to assess the differences between control period, intervention period and follow‐up for each measured variable. Significant differences were observed post‐intervention for sleep efficiency (*p* < 0.001) and sleep latency (*p* < 0.001), whereas the athlete sleep behaviour questionnaire score significantly improved in the follow‐up period (week 9) post intervention (*p* = 0.039). This is the first study to present this novel method of individualised sleep hygiene education for elite female athletes and is also the first study to demonstrate the use of sleep hygiene interventions to improve sleep factors for female athletes' mid‐season. This demonstrates a promising, time‐efficient approach to sleep hygiene education, with a potentially wide scope of application, as well as demonstrating there is indeed potential for elite female athletes to gain sleep improvements mid‐season.

## Highlights

- Sleep hygiene education can be effectively delivered via media messaging to professional female athletes, giving a time‐ and resource‐efficient method of sleep enhancement
- This can improve the sleep of professional female footballers
- Professional female footballers can gain sleep improvements mid‐season

## 1 INTRODUCTION

Sleep hygiene can be defined as practising habits that facilitate sleep and avoiding behaviours that inhibit sleep (Mastin et al., 2006)—it is a simple, non‐invasive, low‐cost strategy which can be used to enhance both sleep quality and sleep duration (SD). Poor sleep hygiene is common among athletes for a variety of reasons such as scheduling constraints and lifestyle factors (Halson, 2019), with Sargent et al. (2021) reporting only 3% of athletes report satisfaction with their sleep. Many aspects of physical performance are positively affected by sleep (Walsh et al., 2021, Mah et al., 2011, Schwartz & Simon, 2015). Costa et al. (2022) stated the importance of sleep for emotional regulation and maintaining overall mental health in athletes, as well as reducing potential illness and injury risk. Despite this knowledge, more than 70% of athletes are regularly operating in a sleep debt (Sargent et al., 2021); therefore, strategies for sleep enhancement must be considered a priority by coaches. Sleep hygiene education is one such method that has been demonstrated to be an effective tool to enhance athletes' sleep (Caia et al., 2018).

O’Donnell and Driller (2017) found that sleep hygiene education resulted in significantly improved total sleep time (mean improvement 22.3 ± 39.9 min), wake variance and wake episode duration for elite netballers. These findings are supported by Caia et al. (2018) and Driller et al. (2019), who both demonstrated sleep hygiene education sessions to be effective for improving sleep factors for elite male rugby players and elite male cricketers, respectively. Despite the vast inter‐ and intra‐individual variation of sleep (Costa et al., 2019), only very few studies have utilised an individualised sleep hygiene education approach within athletic populations. Driller et al. (2019) provided 30‐min individualised sleep hygiene education for 9 male cricketers, during which athletes were given feedback on their baseline sleep measurements, as well as practical tips to resolve any self‐reported issues. In terms of the measured sleep variables pre‐to‐ post, there were significant improvements in sleep efficiency (SE) % (5%, *d* = 1.38, very large), latency (−29 min, *d* = −0.85, large) and sleep onset variance (−28 min, *d* = −0.88, large) following the intervention. In a case study of a male academy footballer, Edinborough et al. (2023) found an individualised sleep hygiene education intervention, based on participant discussion around self‐reported issues, as well as general sleep hygiene advice, to be effective in improving awakening per night (Pre: 7.9 ± 3, Post: 4.5 ± 1.9, −43%) and waking per hour (Pre: 1.2 ± 0.5, Post: 0.6 ± 0.2, −50%), coinciding with an improvement in the athletes' self‐report of Pittsburgh Sleep Quality Index.

Football presents many contextual challenges in relation to sleep, specifically the recurring circumstances of travel, fixture congestion and evening matches, which together are likely to compromise recovery (Fullagar et al., 2016). Furthermore, with the suggestion that match demands for female players vary according to playing position, score line and final result (Martinez‐Hernandez et al., 2024), an individualised approach towards recovery interventions may be warranted. Studies have reported more prominent intra‐individual variation in SE and onset latency in professional footballers, as well as wider athletic populations (Leeder et al., 2012) compared to age‐matched non‐athletic controls (Whitworth‐Turner et al., 2018). Given the amount of individual variation within sleep and lifestyle factors, an individualised approach would seem logical where possible, yet this approach is not commonly used, perhaps due to the potential lack of resources or increased time‐burden on coaches within sporting environments. The cause of such individual variation is likely multifactorial yet commonly overlooked. Indeed, Fullagar et al. (2016) suggested habitual tendencies render the prescription of generic sleep recommendations illogical. Previously, differences have been highlighted between male and female sleep factors, with female sex cited as a risk factor for poor sleep (Walsh et al., 2021); therefore, there is a need for investigations into individualised sleep optimisation approaches for female athletes.

With time pressures on coaches within elite sport high, novel, time‐efficient methods of sleep hygiene education which can also be individualised warrant investigation. Recent research has shown a move towards more novel methods of sleep hygiene education; Hassanin et al. (2023) used sleep hygiene videos (2.44–3.27 min long) with fifth and sixth grade children (*n* = 49) with the cartoon videos designed to educate on the importance of sleep and practical tips. The authors observed a positive change in the Pittsburgh sleep quality index score, 13.6% improvement in SD, 10.9% improvement in sleep disturbance and 22% improvement in sleep latency (SL). Similarly, in a pre‐intervention study, Putri (2023) described the initial trials of sleep hygiene videos (3–6 min long) for the elderly, with all videos delivered via WhatsApp for the reasons of cost and time efficiency. To the authors’ knowledge, no research exists on the development and use of media‐based sleep hygiene delivery to athletes, yet this could provide a practical, time‐efficient method of sleep hygiene education with a broad scope of use. Walsh et al. (2021) highlighted that sleep hygiene education should be delivered multiple times throughout a season, a notion further supported by the evident transient effects of singular bouts of sleep hygiene education in athletes (Caia et al., 2018; Vitale et al., 2019). Therefore, given the lack of time and/or resources was the most cited barrier to sleep monitoring from a range of practitioners working within elite sport (Hough et al., 2021) and that approx. 70% of athletes use their electronic device in the evening (Knufinke et al., 2018),the development of media‐based sleep hygiene education that facilitates repeated exposure to sleep hygiene education with minimal additional coach workload warrants investigation.

Gaps in the literature around time‐efficient, individualised sleep hygiene interventions for professional athletes were identified. Additionally, the lack of research on professional female athletes has previously been highlighted as a key gap in the literature (Paul et al., 2023), which, given females are likely to report a worse sleep status than males (Walsh et al., 2021), led to considering professional female athletes as a primary demographic for this study. Sleep hygiene strategies have previously been used within other athlete populations (Driller et al., 2019) and have been successful in enhancing sleep indices. Liang et al. (2024) noted gamification and technology‐based interventions may increase engagement with sleep hygiene strategies. Therefore, the aim of this study was to ascertain the efficacy of delivering visual media‐based sleep hygiene education (using both animated Graphics Interchange Format (GIF) and single sentence messaging) and whether this could positively affect athletes' sleep indices.

## 2 METHOD

**FIGURE 1:**
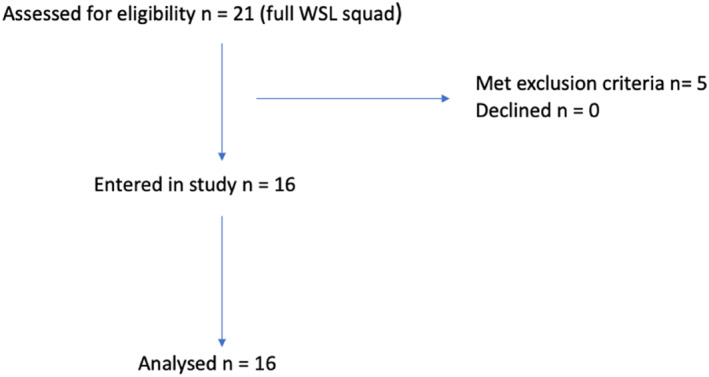
Recruitment process for the study.

Sixteen professional female footballers (age range 21–29 years, mean age 24.4 ± 2.6 years) gave informed consent and volunteered to take part (Figure 1). All participants were currently playing regularly in Women's Super League (WSL), which is the highest level of women's football in the United Kingdom; *n* = 6 were established international players for their respective countries. All players were part of the same club. Full demographics are detailed below in Table 1.

**TABLE 1:** Participant demographics.

Participant demographics		
	Mean	SD
Age (years)	24.4	2.6
Height (cm)	168.3	9.1
Mass (kg)	64.6	10.1
Weekly training hours (football)	10.1	3.9
Weekly training hours (gym based)	3.1	0.6

**FIGURE 2:**
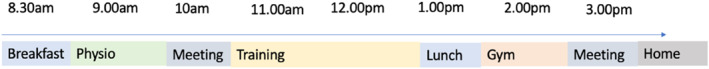
A typical training day schedule for all players within the study, with variations occurring to this usual routine on the days pre‐ and post‐match.

During the study, a total of 8 WSL matches were played, with mean playing time of 68.7 ± 35.9 min. The typical daily schedule for players is presented in Figure 2 below, with variations occurring in the presented schedule on the day pre‐ and post‐match. Individual players varied in the length of time spent in physiotherapy and gym environments, depending on individual needs that week.

Across all participants, *n* = 8 reported regularly taking hormonal contraceptives (type unspecified), whereas *n* = 8 were classified as naturally menstruating women (according to the definition by Elliot‐Sale et al. (2021), as having a menstrual cycle lengths ≥21 days and ≤35 days, but without confirmed ovulation). Prior to the commencement of the study, all participants were informed of study requirements and gave informed consent. Participants were excluded if they reported a pre‐existing sleep disorder, had a current long‐term injury (>1 month), had a menstrual cycle outside the range of 21–35 days or did not give informed consent. Institutional ethical approval was issued (approval number 2023–12896) in accordance with the principles of the Declaration of Helsinki 1964 (revised 2013).

### 2.1 Experimental approach

**FIGURE 3:**
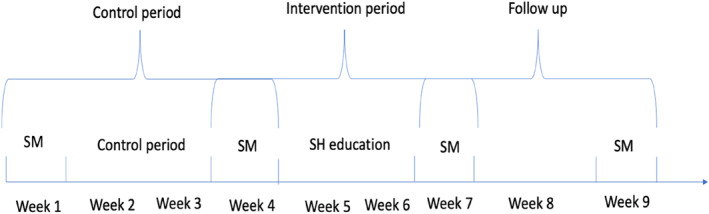
Schematic of the study method detailing the week‐by‐week approach (SM: Sleep monitoring, SH: sleep hygiene).

A self‐controlled time series design, with repeated measures, was used; each participant completed a control period and an intervention period, meaning each participant could act as their own control across the study duration. This approach was taken due to the relatively small sample size available to maximise statistical power (Petersen et al., 2016) and is well suited for hypothesis generation and preliminary testing of novel interventions where a strong evidence base does not yet exist (Brown et al., 2020). A schematic of the study method is presented below in Figure 3.

### 2.2 Sleep monitoring weeks 1, 4, 7 and 9

All participants were allocated an actigraph (GeneActiv Original, Activinsights, Cambridge UK), which they were instructed to wear continuously during monitoring weeks. The device contains a triaxial MEMS‐accelerometer with a range of ±8 g and a sensitivity of ≥0.004 g (te Lindert & Van Someren, 2013). It recorded both motion‐related and gravitational acceleration and has a linear and equal sensitivity along the three axes. Devices were set with a sampling rate of 50 Hz and participants were instructed to wear the device on whichever wrist they felt more comfortable with (Driller et al., 2017). Every week where sleep data was collected, each morning, participants were asked to provide self‐reported sleep quality (Likert scale response) and ‘lights out’ time and wake up time via a Microsoft Forms questionnaire sent by email to each participant. No missing data was identified during monitoring weeks.

All participants completed the athlete sleep behaviour questionnaire (ASBQ) (Driller et al., 2018) to determine current sleep behaviours and sleep hygiene practices. The survey asks participants to rate on a Likert scale how frequently they engage in specific behaviours (never = 1, rarely = 2, sometimes = 3, frequently = 4 and always = 5). Scores were summed to provide an ASBQ global score; higher scores were considered indicative of worse sleep habits and sleep hygiene. The ASBQ has previously been demonstrated to have acceptable test‐retest reliability (ICC = 8.87, coefficient of variation = 6.4%, Driller et al., 2018). In week 1, participants completed the reduced morningness:eveningness questionnaire (rMEQ, Adan & Almirall, 1991), with scores summed to determine chronotype classification as reported in Adan and Almirall (1991): definitely morning type (22–25), moderate morning type (18–21), neither type (12–17), moderate evening type (8–11) and definitely evening type (4–7). The rMEQ has previously been validated against actigraphy (Natale et al., 2006) and has been shown to have good reliability (*α* = 0.71) (Tonetti & Natale, 2019).

### 2.3 Control period week 2 and 3

Participants were instructed to carry on with daily routines as normal. No information was given to participants regarding their baseline sleep data recorded from week 1.

### 2.4 Sleep hygiene education weeks 5 and 6

Baseline sleep data for all participants was reviewed; any item scored above a ‘3 = sometimes’ on the ASBQ was identified as an area for improvement and was reviewed in conjunction with actigraph data to determine the optimal approach for improvement. These identified areas were viewed holistically, as suggested by Mastin et al. (2006), therefore incorporating brief discussions (10 min) with each participant allowed sleep hygiene factors to be viewed in context, with the aim of understanding individual daily routines and habits.

### 2.5 Development of media‐based sleep hygiene education tool

Previous literature commonly adopts a generic approach to sleep hygiene education, with education sessions designed to cover multiple aspects of sleep hygiene, which may or may not be relevant to all participants (Caia et al., 2018; O’Donnell and Driller et al., 2017). The approach to education adopted within this study looked to identify key problem areas from athletes' sleep data and then subsequently develop media‐based messages specific to individual participants areas for improvement. For each participant, target areas of sleep hygiene were identified from the data (via the review of ASBQ data and individual participant discussion around perceived issues, reviewed by the lead researcher who has specific expertise (Doctor of Philosophy) in athlete sleep hygiene), from which a GIF was developed from Adobe Stock Library videos. A suitable video was chosen, which was then converted into a GIF for the purpose of giving a concise, simple message. The message was accompanied by a purposely short text to facilitate a high engagement rate (Hassanin et al., 2023). Where participants had multiple areas for improvement, priority was given towards active behaviours in the evening, in conjunction with knowledge around participants' daily routines and researcher expertise. Prior to the start of the intervention period, a 10‐min group briefing session informed participants that they would be receiving a daily message via WhatsApp for the next two weeks, from which they should try and initiate the action point within the message, that evening. Interpretation of baseline sleep data alongside individual participant discussions presented the following areas for sleep hygiene improvement across all participants (Table 2—*separate file*).

**TABLE 2:** Summary of individualised advice provided to players within the intervention period.

Issue and observation method	Strategy	Content of recommendation (Gif image and wording)	Rationale	Number of participants for which this was used (n)
Technology use before bed (ASBQ)	Screens away 30 min before bed	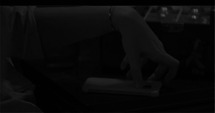 Screens away 30 min before bed.	Limiting phone use 30 min before lights improved sleep duration (Fullagar et al., 2016), reduces sleep latency and improved sleep quality (He et al., 2021). Social media use before bed has a negative effect on sleep quality (Watkins et al., 2022).	15
Poor sleep regularity (ASBQ, actigraph)	Maintain regular bedtimes	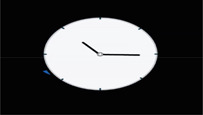 Choose a bedtime and stick to it daily.	Consistency in sleep timing is key for improving sleep efficiency in athletes (Halson et al., 2022). Sleep regularity shown to have positive effects on sleep duration (Phillips et al., 2017).	12
Evening stress, worrying about sports performance (ASBQ, discussion)	Evening stretching routine	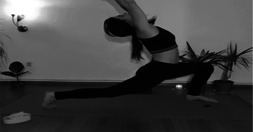 Go through recovery stretches this evening before bed^a^	Stretching in the evening can reduce sleep latency and promote the onset of sleep (Bender et al., 2024; D’Aurea et al., 2019). Short stretching beneficial for reducing anxiety (Montero‐Marin et al., 2015).	9
Poor evening routine and long sleep latency (ASBQ, actigraph, discussion)	Reading in bed before sleep onset	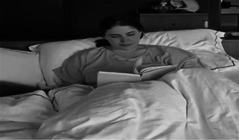 Make reading your final activity before sleeping.	Reading in bed increases melatonin concentration compared to tablet use (Jones et al., 2021) and may improve sleep quality (Finucane et al., 2021).	4
Bedroom environment (ASBQ, discussion)	Maintain a cool, dark and quiet bedroom	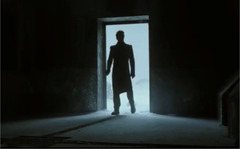 Ensure your bedroom is cool, dark and quiet.	Bedroom environment should be cool (17–23°) (Caddick et al., 2018; Pan et al., 2012) dark (complete blackness) (Caddick et al., 2018) and quiet (<35 dB) (Caddick et al., 2018) to optimise sleep quality (Brown et al., 2002).	3
Evening light exposure (ASBQ, discussion)	Use lamps in the evening	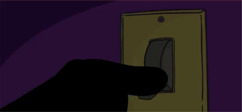 Turn off the main light! Use lamps this evening.	Bright evening light can suppress melatonin secretion, negatively impacting sleep latency and sleep quality (Bender et al., 2024)	2
Evening caffeinated drinks^b^ (ASBQ, discussion)	Replace with non‐caffeinated herbal tea or water	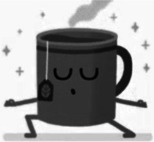 Replace evening caffeine with herbal tea or water.	Caffeine consumption <6 h before bed has disruptive effects on sleep (Drake et al., 2013).	2

^a^ Participants were familiar with a recovery stretching routine aimed at relaxation which was used within their usual training environment as part of the recovery protocol.

^b^ No evening matches were scheduled during the intervention period; therefore, evening caffeine use in this context refers to the habitual consumption of caffeinated drinks as a lifestyle choice rather than a performance‐enhancement effort.

### 2.6 Sleep hygiene message delivery

Each participant was allocated 3 messages based on the interpretation of their individual sleep data; only one was sent per day, which was texted directly to the participant's phone. Another message from their allocation would then be sent the following night and so on. When all their allocation had been used, the first one was sent again in a rotation across the intervention period. The timing of the messages was aimed to optimise the impact with all messages sent in the early evening (8.00 p.m.), giving 2.3 ± 0.65 h between timings of messages and bedtimes. This aimed to give enough time for the participants to action the point and consider the point as part of their evening routine. Technical coaches had informal conversations with participants during the intervention period (during regular scheduled training sessions) to discuss any potential issues and ensure messages were being received—no participants raised any concerns. Coaches continued to verbally encourage adherence throughout the intervention period. Adherence to the intervention was monitored via a subjective report the following morning. Specifically, participants were asked to provide a simple ‘yes’ or ‘no’ as to whether they implemented the suggested action and provide a subjective value of how difficult it was for them to implement the sleep hygiene suggestion the previous night (0 = very difficult to 10 = very easy), as demonstrated by Vitale et al. (2019). This was reported via Microsoft Forms.

### 2.7 Statistical analyses

Descriptive statistics (mean ± SD) were calculated for all variables. Data was checked for normality using Shapiro–Wilk tests and inspection of skewness–kurtosis. Once data met assumptions for parametric statistical analyses, one‐way analysis of variance (ANOVA) with repeated measures was conducted to assess the differences between control period, intervention period and follow‐up for each measured variable. Tukey's Post Hoc tests were used to determine where any potential significance lay between the weeks. Partial Eta squared was reported for ANOVA analysis to give an indication of effect size, with values of 0.01, 0.06 and 0.14 considered as small, medium and large effect sizes, respectively (Lakens, 2013). The data was a median split for each measured variable, with the percentage change presented to demonstrate differences between pre‐ and post‐data for each group plus effect sizes to show the magnitude of change. Threshold effect sizes for percentage changes were considered as follows, based on Hopkins et al. (2009): trivial ≤0.2, small >0.2, >0.6 moderate, and >1.2, ≥2.0 very large.

## 3 RESULTS

A total of 448 observations were taken from 16 participants across a 9‐week period (4 different testing weeks). The chronotype distribution of participants was as follows: definite evening type *n* = 1, moderate evening type *n* = 3, neither type *n* = 7, moderate morning type *n* = 4 and definite morning type *n* = 1. No significant differences were observed between weeks 1 and 4 for any monitored variable (control period).

### 3.1 Adherence and ease of implementation

Total adherence to the intervention was 97% across all participants. Participants graded the ease of following all recommendations 7.00 ± 2.19 on the 10‐point scale. Table 3 below presents the mean ease of the implementation score for each sleep hygiene suggestion.

**TABLE 3:** Mean ± SD participants subjective rating for the ease of implementation of each suggestion on the 10‐point scale (0 being very difficult, 10 being very easy).

Sleep hygiene suggestion	Mean ease of implementation score	Standard deviation
Screens away 30 min before bed	5.17	0.65
Maintain regular bedtimes	4.06	1.11
Evening stretching routine	8.64	0.6
Reading in bed before sleep onset	8.52	0.79
Maintain a cool, dark, quiet bedroom	5.1	0.58
Use lamps in the evening	9.29	0.86
Replace evening caffeine with alternatives	8.35	0.86

Abberivation: SD, sleep duration.

**FIGURE 4:**
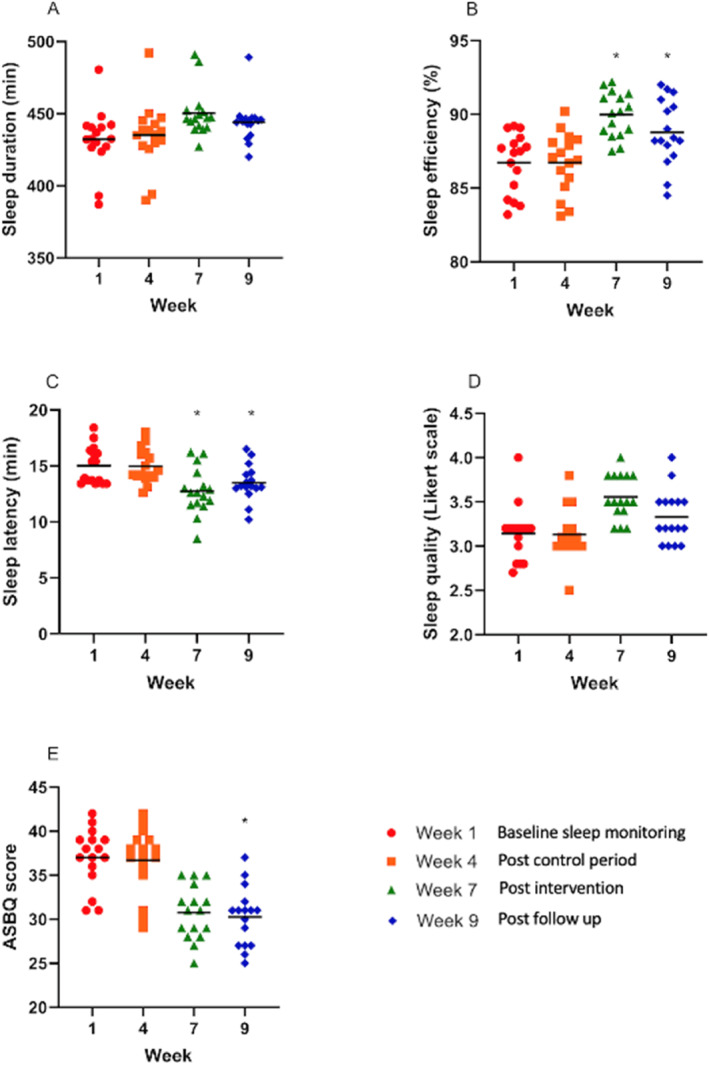
A–E: Scatter plots indicating the spread of data and means for each measured variable. *p < 0.05 for inter‐week differences from the control period to post‐intervention. Plot C and E, a decrease in mean value indicates an improvement than the indices.

Figure 4 below presents individual and mean data for each measured variable.

In terms of significantly improved sleep measures, SE significantly increased between weeks 4 and 7 (F (3, 444) = 10.2, *p* < 0.001, ηp^2 =^ = 0.12) and between weeks 4 and 9 (*p* < 0.001). SL significantly decreased (indicating a more favourable SL) between weeks 4 and 7 (F (3, 444) = 13.6, *p* < 0.001, ηp^2 =^ = 0.08) and between weeks 4 and 9 (*p* < 0.001). Self‐reported ASBQ scores significantly decreased (indicating more favourable sleep behaviours) between weeks 4 and 9 (F (3, 60) = 5.69, *p* = 0.039, ηp^2 =^ = 0.22).

**FIGURE 5:**
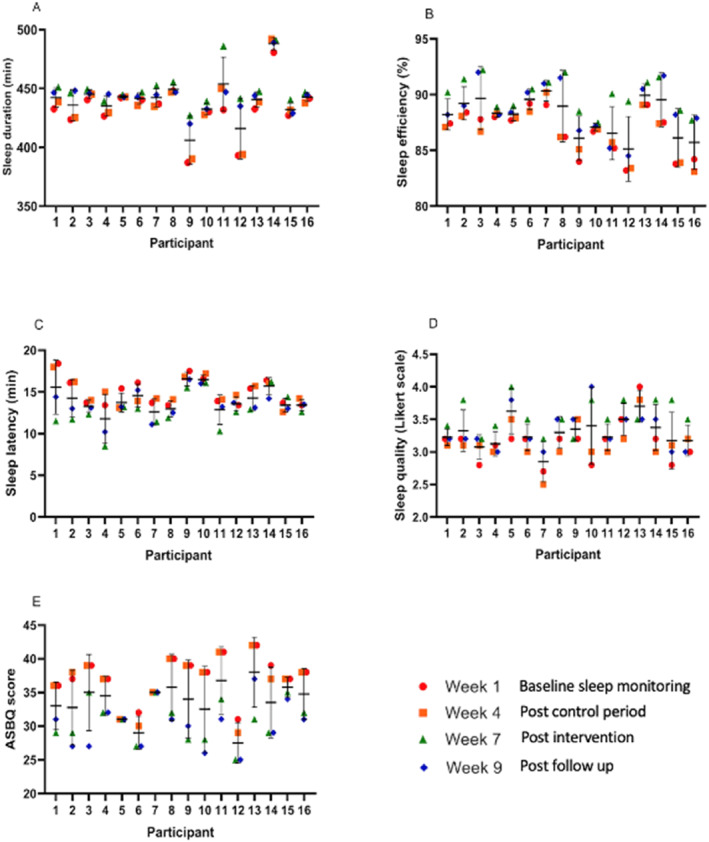
Individual data plots for each measured variable.

The median split of data for each measured variable demonstrated those starting with lower baseline scores across all variables and showed a greater percentage change following the intervention period (Figure 5 and Table 4).

**TABLE 4:** Median split data percentage changes for the upper and lower halves of the data across all variables following the intervention period and follow‐up period.

	Median group	Week 4‐7 (%)	Effect size	Week 7‐9 (%)	Effect size
Sleep duration	Upper	5	1.0, moderate	−0.1	0.2, trivial
	Lower	12	1.4, large	−1.3	0.6, small
Sleep efficiency	Upper	2	0.5, small	−0.9	0.1, trivial
	Lower	16.3	3.1, very large	−1.6	0.6, moderate
Sleep latency	Upper	16.8	1.6, large	−11	0.9, moderate
	Lower	19.7	1.8, large	−2.9	2.4, very large
Sleep quality	Upper	3.1	0.3, small	−0.3	0.1, trivial
	Lower	2.1	0.2, small	−0.9	0.1, trivial
ASBQ	Upper	3.1	0.4, small	1.9	0.3, small
	Lower	20.9	5.3, very large	5.4	0.6, moderate

For all variables across the intervention period apart from sleep quality, the lower split of participants demonstrated greater percentage improvements than the upper split.

## 4 DISCUSSION

The aim of this study was to ascertain the efficacy of delivering media‐based sleep hygiene education (using both animated GIF and single sentence messaging) and whether this could positively affect female athletes' sleep indices. The main findings were that using media‐based sleep hygiene education across a 2‐week period had significant positive effects on SE, latency and ASBQ scores. Therefore, initial evidence highlights a promising strategy for an individualised sleep hygiene education intervention, demonstrating a time‐efficient method of improving the sleep of professional female footballers during mid‐season. Furthermore, in terms of practical application, this strategy may provide the opportunity for athletes to access specialist advice in sleep without the potential constraint of resources or location for both parties.

Visual media, to portray an educational message, has several advantages, namely that the message conveyed is packaged attractively so that it will be easily remembered by the audience, is not limited by distance or time, and can be repeated (Maramis, 2013). Tuong et al. (2014) showed that media interventions were variably effective in modifying health behaviours depending on the target behaviours to be influenced—results of the present study suggest sleep behaviours may indeed be modifiable by visual media interventions, with female athletes in the present study demonstrating improvements in SE (week 4–7 improvement +2.9%), SL (week 4–7 improvement −3.6 min) and ASBQ score (week 4–9 improvement −4.2 reduction on the ASBQ score). This concurs with Asih et al. (2024), who concluded sleep hygiene education, delivered via an animation video as a first‐line strategy, to be beneficial in improving sleep hygiene practices, although it should be noted that Asih et al. (2024) investigated a different population (pregnant women, *n* = 108). Similarly, Nisa et al. (2021) advocated the use of WhatsApp messaging to increase knowledge and attitudes around sleep among adolescents (*n* = 82, 40 intervention, 42 control). To the authors’ knowledge, this study is the first to apply this novel method of sleep hygiene education to an athlete population.

Previous ease of implementation to sleep hygiene interventions has been reported as 6.35 ± 2.7 on the 10‐point scale by Vitale et al. (2019). In comparison, the present study reports a subjective grading of 7.00 ± 2.19, suggesting the messages used in the present study may be easier to implement than the combination of in‐person education and leaflet used by Vitale et al. (2019). Adherence and ease of implementation are not commonly reported in sleep hygiene studies, with Harada et al. (2016), the only other study to present similar information, reporting an intervention index to sleep hygiene education, which correlated to improvements in football performance (*r* = 0.42, *p* = 0.0003). Reporting this measure may be of value in the practical application of the work, giving reassurance to practitioners regarding the efficacy of such approaches; indeed, Li et al. (2024) stated that any recovery approach is only viable with successful athlete engagement, which is likely to be higher if an athlete perceives that the intervention is relatively easy to implement.

Jenkins et al. (2021) concluded that the delivery of generalised, group‐based sleep hygiene education mid‐season had no effect on sleep parameters for professional rugby players; the authors alluded that sleep hygiene education may only be useful during times in the season with lower stressors, such as pre‐season. Similarly, comparable studies demonstrating positive results of sleep hygiene interventions were also conducted pre‐season (Driller et al., 2019; O’Donnell & Driller, 2017). To the authors’ knowledge, Caia et al. (2018) is the only study demonstrating positive effects on sleep hygiene delivery in mid‐season for professional athletes (rugby), although this was a male cohort. Given the differences in sleep between males and females, both in terms of sleep indices and sleep hygiene factors (Silva et al., 2019), it could be postulated that differences between sexes may be evident in the response to sleep hygiene interventions. It should also be noted that the approach used by Caia et al. (2018) was a median split, meaning those who slept less and had worse sleep habits received sleep hygiene education, while those who slept more received no sleep hygiene education (control group). While this approach is logical from a practical perspective (as individuals with a worse sleep status may benefit more from improvement), the absence of a balanced control group means this may not give an accurate representation of the effectiveness of sleep hygiene education mid‐season. Furthermore, that approach may ultimately lead to different long‐term benefits across a team. The present study adds to the knowledge around the potential usefulness of sleep hygiene education mid‐season for professional athletes, demonstrating an alternative, effective method and is the first study to present this information for professional female athletes. Therefore, coaches are encouraged to consider sleep enhancement strategies for female athletes' mid‐season, with the present study demonstrating the application of media‐based sleep hygiene education to be an effective method of doing so.

Electronic device use in the evening may serve as a distraction before sleep, potentially resulting in the device being used into usual bedtimes (Jones et al., 2021), and this factor should be considered around the timing of media‐based sleep reminders to remove the risk of the intervention becoming a counterproductive in confounding a common issue for problematic sleep. Indeed, Monma et al. (2023) reported 77% of judo athletes (mean age 22.9 ± 3.1) used their phones in bed after lights out, with a prevalence of poor sleep 40.7%. In the present study, messages were sent at a standardised time of 8.00 p.m., which aimed to allow time for actioning the message while also being cognisant of the potential for negative effects of phone use closer to bedtime. In a larger cohort, the time of messaging could be individualised further to be more impactful with the potential for automated messaging to be set up for each athlete from a centralised system, ensuring this remains a simple and viable method for coaches of larger groups. However, given the success of the intervention in the present study with messages sent 2.3 ± 0.65 h pre‐bedtime, practical recommendations would be for coaches to adopt similar timings for group‐based delivery.

When looking at the data median split, it is interesting to note that participants within the lower scoring group across all variables apart from self‐reported sleep quality had the greater magnitude of improvement compared to those in the upper group (Figure 4, Table 4). Dunican et al. (2023) delivered sleep hygiene education to recreational swimmers (*n* = 11 male, *n* = 13 female) and found no significant differences in sleep pre‐post. The authors alluded to a potential ceiling effect for sleep hygiene education, given that the pre‐test data showed sleep factors that were close to optimal recommendations. This may explain the difference in the magnitude of improvements in the present study, with those starting from a lower baseline having greater potential for improvement than those who were already close to recommendations. These results support the previous postulation from Vitale et al. (2019), who suggested sleep hygiene interventions may be of greater benefit for athletes reporting poor baseline sleep behaviours. Poor baseline sleep habits have been regularly reported within football cohorts (Fullagar et al., 2016; Nedelec et al., 2015) with cultural constraints around sleep, such as variable sleep timings, match outcome‐related stress and match timings, suggested as negative influences on sleep habits (Nedelec et al., 2015), which are likely to be repeated stressors throughout the season. Considering this, it is likely that many players may benefit from such an intervention as presented within this study.

During the follow‐up period (week 9), results showed a decrease in SD (5 min decrease), efficiency (1% decrease), latency (6 min increase) and quality (compared to week 7), although SD, efficiency and latency were still improved from baselines. Sleep hygiene interventions have commonly been shown to have transient improvements; Caia et al. (2018) found sleep indices were comparable to baseline 1‐month post sleep hygiene education for professional male rugby players. This notion is further demonstrated by Vitale et al. (2019) who implemented a 45‐min group‐based sleep hygiene session prior to an evening football match, with sleep factors monitoring 1‐ and 2‐night post‐intervention. The authors demonstrated comparable sleep factors to baseline 2 days post sleep hygiene intervention. Results of the present study suggest a slower decay effect than Vitale et al. (2019), likely due to methodological differences, with Vitale et al. (2019) implementing a singular bout of sleep hygiene education. This could infer the use of repeating sleep hygiene messages to be of greater benefit for maintaining sleep improvements than singular inputs. The sleep hygiene strategy used within the present study is conducive to intervention ‘top ups’ and thus a longitudinal study over greater duration (i.e., over a season) may be relevant in determining an intervention period followed by a ‘maintenance period’ approach.

## 5 LIMITATIONS AND FUTURE RESEARCH

Sample size was limited in this study due to athlete availability and overall squad size, and the study would benefit from being repeated with a larger subject group to increase statistical power. The method of sleep hygiene education used in this study means the success of the intervention cannot be attributed to a single change in athlete behaviour; therefore, further research is suggested to determine the level of impact each message may have. This would allow for a more targeted, and potentially more efficient and effective, approach to be implemented. Additionally, future research should aim to determine a minimal dose response. Investigating athletes from different sports and within different environments would be beneficial to understand the efficacy of this strategy among different cohorts, while a longer intervention and follow‐up period may be of interest to determine whether effects can be sustained throughout a season. As there was some evidence of a decay effect following this intervention, determining the amount of ‘top ups’ required to sustain improved sleep factors would be useful in presenting more detailed practical guidance. Finally, further research should look to conduct the study with a sample size suitable to determine whether chronotype preferences affect responsiveness to the intervention—this study did not meet minimum sample size requirements to run multiple regression analysis to determine this (Jenkins & Quintana‐Ascencio, 2020), yet it would be of value within the process of athlete screening and monitoring, and in order to potentially prioritise intervention.

## 6 CONCLUSIONS

In conclusion, this study is the first to present a novel, media‐based method of sleep hygiene education for professional female athletes, which has shown to have significant positive effects on SE, latency and ASBQ scores across a 2‐week intervention period during mid‐season. This demonstrates a promising, time‐efficient approach to sleep hygiene education with a potentially wide scope of application. A potential future hypothesis for further investigation would be that media‐based sleep hygiene delivery to professional athletes is more effective on improving a variety of sleep factors than in‐person delivery due to the repeated nature and the timing of the delivery.

## CONFLICT OF INTEREST STATEMENT

The authors declare no conflicts of interest.

## ETHIC STATEMENT

Ethical approval was sought from all participants prior to commencing the study. Institutional ethical approval was issued from the University of Brighton, UK (approval number 2023‐12896‐Gooderick) in accordance with the Declaration of Helsinki 1964 (revised 2013).

## Data Availability

Data is available from the lead author, Julie Gooderick, upon reasonable request.
